# Engagement With Digital Health Technologies Among Older People Living in Socially Deprived Areas: Qualitative Study of Influencing Factors

**DOI:** 10.2196/60483

**Published:** 2024-12-26

**Authors:** Helen Chadwick, Louise Laverty, Robert Finnigan, Robert Elias, Ken Farrington, Fergus J Caskey, Sabine N van der Veer

**Affiliations:** 1 Division of Informatics, Imaging and Data Sciences, Manchester Academic Health Science Centre, The University of Manchester, Vaughan House, Portsmouth Street, Manchester, M13 9GB, United Kingdom, 44 1613067767; 2 NHS England North West Kidney Network, Manchester, United Kingdom; 3 King's Kidney Care, Kings College Hospital NHS Foundation Trust, London, United Kingdom; 4 Centre for Health Services and Clinical Research, The University of Hertfordshire, Hatfield, United Kingdom; 5 Bristol Medical School, University of Bristol, Bristol, United Kingdom

**Keywords:** aged, digital health, health equity, intersectionality, qualitative research, social deprivation

## Abstract

**Background:**

The potential benefits of incorporating digital technologies into health care are well documented. For example, they can improve access for patients living in remote or underresourced locations. However, despite often having the greatest health needs, people who are older or living in more socially deprived areas may be less likely to have access to these technologies and often lack the skills to use them. This puts them at risk of experiencing further health inequities. In addition, we know that digital health inequities associated with older age may be compounded by lower socioeconomic status. Yet, there is limited research on the intersectional barriers and facilitators for engagement with digital health technology by older people who are particularly marginalized.

**Objective:**

This study aimed to explore factors influencing engagement with digital health technologies among people at the intersection of being older and socially deprived.

**Methods:**

We conducted semistructured interviews with people who were 70 years or older, living in a socially deprived area, or both. Chronic kidney disease was our clinical context. We thematically analyzed interview transcripts using the Unified Theory of Acceptance and Use of Technology as a theoretical framework.

**Results:**

We interviewed 26 people. The majority were White British (n=20) and had moderate health and digital literacy levels (n=10 and n=11, respectively). A total of 13 participants were 70 years of age or older and living in a socially deprived area. Across participants, we identified 2 main themes from the interview data. The first showed that some individuals did not use digital health technologies due to a lack of engagement with digital technology in general. The second theme indicated that people felt that digital health technologies were “not for them.” We identified the following key engagement factors, with the first 2 particularly impacting participants who were both older and socially deprived: lack of opportunities in the workplace to become digitally proficient; lack of appropriate support from family and friends; negative perceptions of age-related social norms about technology use; and reduced intrinsic motivation to engage with digital health technology because of a perceived lack of relevant benefits. Participants on the intersection of older age and social deprivation also felt significant anxiety around using digital technology and reported a sense of distrust toward digital health care.

**Conclusions:**

We identified factors that may have a more pronounced negative impact on the health equity of older people living in socially deprived areas compared with their counterparts who only have one of these characteristics. Successful implementation of digital health interventions therefore warrants dedicated strategies for managing the digital health equity impact on this group. Future studies should further develop these strategies and investigate their effectiveness, as well as explore the influence of related characteristics, such as educational attainment and ethnicity.

## Introduction

The World Health Organization (WHO) defines digital health technologies as “the field of knowledge and practice associated with the development and use of digital technologies to improve health” [1]. The digitization of health care services, expedited by the onset of the COVID-19 pandemic in 2020, has been ongoing for decades [2]. This means health care providers and patients worldwide increasingly use digital health technologies, such as web-based consultation platforms [3], that need accessing via a website or mobile app.

On one hand, the benefits of digital health care are well documented; for example, patients report feeling empowered to be an active participant in their own health care through having access to web-based health records and digital tools for health management [4]. In addition, the use of digital health technologies can improve access for patients with limited mobility, such as patients with physical disabilities and frail older adults, and those living in remote or underresourced locations [5]. On the other hand, however, moving toward a health care system that is increasingly digitized means that not everyone will have equal access to the benefits it can provide. People with the greatest health needs, including those who are older or living in more socially deprived areas, are often least likely to have access to digital technologies and may not have the skills to use them [6,7]. The effects of the COVID-19 pandemic demonstrated that poverty, lack of access to digital health technologies, and poor engagement with digital health contribute to poor health outcomes [8]. This means that these already disadvantaged groups are often most at risk of (further) digital health inequities [7,9].

Literature reviews have investigated key factors that influence engagement with eHealth interventions in populations with lower socioeconomic status [10], as well as in older adults [11]. However, we know that digital health inequities associated with older age may be compounded by lower socioeconomic status [12]. Yet, there is limited research on the intersectional practical and conceptual barriers and facilitators to engagement with digital health technology by groups of older people who are particularly marginalized [11,13]. We need to better understand these barriers and facilitators to inform successful digital health implementation strategies and avoid perpetuating health inequities for disadvantaged patient populations [14].

Therefore, this study aims to explore factors that influence engagement in digital health technology by older people and those living in socially deprived areas, with a particular focus on the intersection between these 2 characteristics. We used chronic kidney disease (CKD) as the clinical context because older people and people from areas of greater deprivation are at risk of kidney health inequalities [15], and there is limited research considering the factors that influence engagement with digital health technology in these populations [16]. Ultimately, this will help inform successful and equitable digital health implementation strategies in CKD and other long-term conditions.

## Methods

### Study Design

We conducted a qualitative descriptive study involving semistructured, individual interviews with people with kidney disease, which we reported in line with the Consolidated Criteria for Reporting Qualitative Research (COREQ) [17] (Supplement 1 in Multimedia Appendix 1 provides a completed checklist).

### Ethical Considerations

The study was reviewed and approved by the Northwest (Liverpool Central) Research Ethics Committee (reference 22/NW/0127). All participants provided written informed consent after receiving a participant information sheet and having an opportunity to ask questions. They received £20 (US $25) in shopping vouchers to thank them for their time. Interview transcripts were deidentified and stored with participants’ study IDs and separated from their personal information. Quotes included in this manuscript were anonymized by removing any information that might identify individual participants.

### Theoretical Framework

We used the Unified Theory of Acceptance and Use of Technology (UTAUT) [18] to guide our study design. The UTAUT aims to explain people’s intended and actual adoption of technology. It consists of 4 constructs: performance expectancy (beliefs that using the technology will help); effort expectancy (degree of ease of using the technology); social influence (degree to which a person perceives that important others believe they should use the technology); and facilitating conditions (degree to which a person believes that support exists to facilitate the use of the technology). It also proposes 4 moderators (gender, age, experience, and voluntariness of use). Several previous studies used the UTAUT to understand potential barriers and facilitators to engage with digital health technologies [19-21].

### Study Setting

We conducted our study in the context of hospital-based kidney centers that deliver secondary health care services for people with CKD in England (United Kingdom) via outpatient clinics, as well as via hemodialysis units.

### Eligibility and Recruitment

Local research nurses identified and approached people with CKD from 3 kidney centers by screening outpatient clinics and dialysis unit patient lists. The research team additionally recruited patients via web-based local and national patient groups (including the Kidney Patient Involvement Network, Kidney Information Networks, the Renal Patient-Led Advisory Network, and Kidney Care UK). We sent the study flyers and participant information sheets (including a section on “What is the purpose of the research”) to potential participants via post or email. We did not keep a record of how many people refused to participate.

People were eligible if they (1) were an adult (≥18 years) with CKD, (2) were 70 years of age or older and had a postcode within deprivation deciles 1-3 (indicating an area of high relative socioeconomic deprivation based on the Index of Multiple Deprivation [IMD]) [22], and (3) were able and willing to provide written consent, and able to understand and speak English.

We purposively recruited participants who had both characteristics, as well as those who were just older or just socially deprived. This allowed us to explore whether certain factors were specific to being at the intersection or if they also became apparent in people having only one of the characteristics.

### Data Collection

One researcher (HC) conducted semistructured interviews between July 2022 and February 2023 via telephone; each participant was interviewed only once. The researcher was female, had an MSc in Health Psychology, worked as a postdoctoral research associate at the University of Manchester, had significant experience in qualitative research, and had completed good clinical practice training. The researcher was based in a digital health research group at the University of Manchester (UK). No relationship was established between the researcher and participants prior to the interviews taking place.

We assessed the digital literacy and the health literacy of participants using the Computer Proficiency Questionnaire [23] and the Short Literacy Survey [24], respectively. These were administered either via a web-based survey-hosting platform or over the telephone depending on participants’ preferences.

We developed the interview topic guide based on our theoretical framework [18] and pilot-tested it with our kidney patient partner (RF) (Supplement 2 in Multimedia Appendix 1). It included topics related to participants’ perspectives on familiarity with digital health technologies; access to digital health technologies; perceived advantages and disadvantages of digital health technologies; and barriers or facilitators to engagement with digital health technologies. We iteratively refined the topic guide over the course of the data collection period based on preliminary findings.

The interviewer took field notes and audio-recorded all interviews, with audio recordings transcribed verbatim by a professional transcription service. We did not share transcripts or findings with participants for feedback. Data collection ended when subsequent interviews did not add new information compared with previous interviews.

### Data Analysis

We performed a thematic analysis of the interview transcripts using NVivo (version 12; QSR International) qualitative data analysis software. The analysis was primarily deductive, guided by our theoretical framework [18] and the research objectives. However, we took an inductive approach to analyzing the parts of the data that were not directly related to any of the UTAUT concepts. We used the UTAUT concepts as parent codes (ie, performance expectancy, effort expectancy, social influence, enabling conditions, and behavioral intention) and developed additional parent codes for data that did not relate to UTAUT concepts.

The primary coder (HC) developed a preliminary codebook from a first pass at the data, with a second coder (LL) reviewing it to inform further refinements. We then recoded the transcripts using the refined codebook (Supplement 3 in Multimedia Appendix 1 shows the final version). Once all transcripts had been coded, the 2 coders (HC and LL) and a third member of the research team (SNVDV) collectively identified themes and developed them iteratively through several in-depth discussions. During these discussions, we considered whether the themes were unique, whether the data supported the themes, if the themes needed to be broken down further, and whether additional themes needed to be added. We also kept a log of theme additions, modifications, and developments using Microsoft Excel.

## Results

### Participants

In total, we interviewed 26 people, with interviews lasting between 9 and 53 minutes. Some interviews were relatively short due to participants giving brief answers to interview questions and not being able or willing to elaborate when prompted. Table 1 shows the participants’ characteristics. A total of 13 participants were 70 years of age or older and living in a socially deprived area, and the majority were White British (n=20) and had moderate health and digital literacy levels (n=10 and n=11, respectively).

**Table 1.** Demographic characteristics of study participants (n=26).

Characteristics	Value
Sex, n (%)	
Male	14 (54)
Female	12 (46)
Age range (years)	29‐84
Age (years), n (%)	
≥70	24 (92)
<70	2 (8)
Ethnicity, n (%)	
White/White British	20 (77)
Black (British, African, Caribbean)	5 (19)
British Indian	1 (4)
CKD^a^ treatment modality, n (%)	
Hemodialysis	18 (69)
Transplant	4 (15)
Peritoneal dialysis	2 (8)
Missing^b^	2 (8)
Social deprivation, n (%)	
IMD^c^ deciles 1‐3 (most deprived)	16 (62)
IMD deciles 4‐10	10 (38)
Health literacy^d^, n (%)	
3‐7 (lowest health literacy)	3 (12)
8‐12	10 (38)
13‐15	8 (31)
Missing^e^	5 (19)
Digital literacy^f^, n (%)^g^	
6‐15 (lowest digital literacy)	4 (15)
16‐25	11 (42)
26‐30	7 (27)
Missing^e^	4 (15)

a CKD: chronic kidney disease.

b Missing because not recorded by recruiting kidney center staff.

c IMD: Index of Multiple Deprivation [22].

d As measured by the Short Literacy Survey [24]; scores could range from 3 to 15, with lower values indicating lower health literacy levels.

e Missing because some participants did not return their questionnaire or did not respond to requests to conduct the questionnaire over the telephone.

f As measured by the Computer Proficiency Questionnaire [23]; scores could range from 6 to 30, with lower values indicating lower digital literacy levels.

g Percentages do not add up to 100% due to rounding.

### Theme 1: Lack of Engagement With Digital Technology in General

#### Summary

Some participants were not or very little engaged with using digital technology of any kind in their daily lives. These participants lacked the necessary skills, resources, and support to engage with digital technology in general. The following sections describe this in further detail, with Supplement 4 in Multimedia Appendix 1 showing all participant quotations.

#### Lack of Opportunity in the Workplace to Become Digitally Proficient

Participants who were less digitally able typically did not have any experience of using digital technology in the workplace. Some older participants explained that they went into retirement before their workplace started to switch over to digital systems, meaning that they missed the opportunity to learn new digital skills. Participants who had previous experience using digital technology, especially in the context of the workplace, reported being more confident and skillful with digital technology. Participants who had previously done jobs that did not involve using a computer (eg, manual labor and jobs that were not office-based) tended to be less digitally proficient.

*Because my career was in education and I was running a national government agency, so even at the very early stages, we started having computers and things like that. So I suppose I got quite used to the systems*.

[participant 1; 75 years; IMD: 4]

#### Lack of Access to Digital Technology and Infrastructure

Most participants who never used digital technology for their health or care often did not have access to the technology itself. Some participants did not own a smartphone device or computer, had poor mobile phone reception, or did not have access to a (good) internet connection at their home address.

*I haven’t got it on my phone, the internet…I’ve never had a computer*.

[participant 13; 73 years; IMD: 1]

For some participants, especially those living in socially deprived areas, financial instability meant that they had limited funds to spend on digital technology. Several participants mentioned that the rising costs of living meant that their ability to pay for their monthly internet was becoming precarious.

*Yeah, we have problems with money, yeah. We struggle paying for things. If you haven’t got money to pay the internet, don’t have it. My husband says, if we haven’t got the money this month, we won’t pay it*.

[participant 12; 48 years; IMD: 3]

#### Lack of Support to Use Digital Technology

Where participants had little or no access to digital technology, this was exacerbated by the fact that in most cases, their health care provider had not offered support for using digital health technology. Numerous participants therefore needed one-on-one guidance from their family members to be able to use and benefit from digital technology; some did not have any family living nearby or who had the capacity or patience to provide support when needed. In most cases where support with digital technology was provided by family members or friends, the supporter did not tend to take on a teaching role. Instead, the supporter would simply complete the task, leaving the participant with a missed opportunity to learn any new digital skills.

*I do have a friend who lives in the next area and she would come and help me, but she can’t come straight away. It’s difficult*.

[participant 19; 75 years; IMD: 1]

*I thought my grandson would probably, but I don’t think he’d be a good teacher. I think he’d just snatch the phone, do it and look at me witheringly*.

[participant 17; 76 years; IMD: 2]

Despite there being a general lack of awareness of and access to community/charity-run digital support, several participants said that they would be willing to attend educational classes to improve their digital skills if they were available to them.

### Theme 2: Digital Health Technology Is “Not for Me”

#### Summary

Many participants expressed total disinterest in engaging with digital technology for the purpose of monitoring or improving their health. They were unmotivated to use digital health technologies because they perceived these to have no place in their lives. This perception is related to internal factors about personal capabilities and identity, as well as external factors about the nature of digital health technology.

#### Self-Concept of Being “Too Old” to Engage With Digital Health Technology

Participants commonly expressed the notion that they lacked the capacity to “keep up,” mentally and physically, with modern-day technology due to their older age, regardless of their socioeconomic status. They expressed the idea that it was too late for them to learn new digital skills at their age, and that they would not even want to attempt to use digital health technology because they expected to be unsuccessful.

*I don’t have any of that, because I’m a pensioner…I can’t be bothered with all that*.

[participant 10; 81 years; IMD: 5]

For some participants, this was associated with anxiety about using digital health technology incorrectly or making mistakes while using it, sometimes following previous negative experiences. They described their lack of digital capability as being child-like, suggesting a feeling of helplessness and infantilization. This feeling was more commonly expressed by older people who were living in areas of higher social deprivation.

*I think age comes into it. I think age…I don’t have the, I use the word properly, a sense of careless confidence that a child has, but I’ve still got a fear that I’m going to break it*.

[participant 17; 76 years; IMD: 2]

*I’m not very confident and if I was on my own in the house with an online system and it went wrong, I wouldn’t know what to do, I’d be in a terrible state, like a child*.

[participant 19; 75 years; IMD: 1]

#### Self-Monitoring of Health Using Digital Technology Is Burdensome

When asked if they used any digital systems to self-monitor their health remotely, numerous participants explained that they preferred to leave the responsibility of monitoring their health to their doctor. There was a sense that digital health puts the onus on the patient to monitor their own health, whereas traditional health care feels more like being looked after. Some participants felt that keeping track of their health and kidney disease using digital technology was anxiety-inducing, with over-exposure to information about physical health having a negative impact on their mental health.

*I don’t want to see my records…I don’t think that’s very healthy, although a lot of people say, oh, yeah, I wanna know what’s wrong…No, no. I don’t*.

[participant 2; 72 years; IMD: 6]

Many participants had a high disease burden associated with older age, chronic illness, and competing physical or cognitive morbidities. Furthermore, some participants explained that poor finger dexterity, general mobility issues, and poor eyesight made it difficult for them to use digital technologies, such as mobile devices.

*I can’t even use my hands, I can’t hold onto anything now. I can’t hold anything, even my mug so why bothe*r?

[participant 9; 72 years; IMD: 3]

Others explained that cognitive limitations, such as poor memory, made it burdensome for them to engage with digital health solutions for the purpose of self-monitoring. For example, many participants struggled to remember login details for digital health technologies, such as web-based patient portals.

#### Interfaces of Digital Health Technologies Are Not User-Friendly

Many participants communicated that digital technologies were not adequately meeting their health care and technology user needs. Key concerns were that user interfaces were too complicated to navigate without one-on-one support, they did not accommodate complex physical and cognitive needs, and presented content that was too generic.

Some participants found that the processes for setting up and using digital health technologies were not user-friendly or intuitive, which caused frustration and confusion, which ultimately put people off engaging with these technologies. They also noted that patient portals and other digital health technologies were often not fit for purpose. For example, several participants found these difficult to navigate, with information being difficult to access and understand. In addition, several participants complained about receiving too many notifications from patient portals, or not being given the health information that mattered to them.

*I don’t need to know every week that I haven’t got HIV. Nor do I need to know every day how much ferritin I’ve got in my body*.

[participant 17; 76 years; IMD: 2]

#### Rejection of the Benefits of Digital Health Technologies

Some participants felt that digitization of health services was threatening to replace traditional face-to-face appointments, and that this would ultimately result in a reduced standard of care. A number of participants expressed a preference for in-person appointments over digital health care, as they found digital appointments to be cold, impersonal, and insufficient to properly assess health or well-being. The majority of participants expressing these concerns were older and living in areas of high social deprivation.

*I just think if everything is digital, you’re just losing the human touch a little bit more, do you know?*

[participant 6; 71 years; IMD: 2]

*I would see somebody face-to-face because what I don’t like about video calls is that if you were on screen, I wouldn’t know where you were looking. I know it sounds silly, but I don’t know. There’s no connection*.

[participant 17; 76 years; IMD: 2]

There were also concerns that companies providing digital health technologies, such as health apps, were primarily profit-motivated and not necessarily concerned with improving patients’ experience or the standard of care.

## Discussion

### Summary of Findings

This study explored factors that may influence engagement in digital health technologies by older people and those living in socially deprived areas. The first theme suggested that some individuals simply do not engage with digital technology of any kind, with subthemes describing how some participants lacked the necessary skills, resources, and support to engage with digital technology. Participants who lacked digital skills often also mentioned they had not had the opportunity to develop these capabilities in the workplace. In addition, many participants did not receive adequate support to learn how to use digital technology for health care purposes. Both these latter factors were commonly coupled with being older and socially deprived.

The second theme posed the idea that participants were reluctant to engage with digital health technology because of a long-standing belief that it could not be integrated into their daily lives. The subthemes suggested participants believed that they were too old to engage with digital health technology, perceived digital self-monitoring of health as a burden, and rejected the potential benefits of digital health technologies. Of the participants who felt they were too old to use digital health technologies, those who were more socially deprived described feeling anxious about using digital technology incorrectly, which was sometimes accompanied by a sense of helplessness. It was also a belief among the older and more socially deprived participants that digital health care is cold, impersonal, and insufficient to properly assess health or well-being.

### Relation to Other Studies and Theory

Previous studies indicated that multiple layers of disadvantage influence engagement with digital health technologies. For example, underuse of digital health tools in older people was associated with socioeconomic disparity [25], and older adults with higher levels of income and education tended to have better eHealth literacy [26]. However, despite this established connection between older age, social deprivation, and lack of engagement with digital health technologies, there is limited research on what facilitates engagement for this group [13]. Our findings suggested that there are specific factors that influence engagement with digital health technology at the intersection of social deprivation and older age. Although we did not collect demographic data on participants’ employment status and occupational history for all participants, the interview data suggested that, for example, individuals who reported having had manual or “low-skilled” jobs that did not involve using digital technology were typically less digitally able than those who had worked in office-based roles. This aligns with data suggesting that the workforce digital skills gap is particularly pronounced in older people and socially deprived populations [27].

Another example is that numerous older participants who had lower social deprivation scores explained that they lacked friends or family members who were available or had the capacity to help them learn new digital skills. This is in keeping with research suggesting that older people who do not have family members who live with or near them are far less likely to get the necessary level of support they need to engage with digital technology [28], while older residents of deprived urban areas who have a long-term condition are more likely to live alone [29].

We also found that some of our research participants were reluctant to engage with digital health technology because it conflicted with their core beliefs about themselves. Negative self-perceptions around being “too old” to use digital technology were underpinned by feelings of fear about doing something wrong and of inadequacy about lack of digital skills, which is a common finding in literature around the age-based digital divide [30]. These factors align closely with other studies that also used the UTAUT to conceptualize “facilitating conditions” that impact the uptake of digital health interventions in older people and those of lower socioeconomic status [19,20]. These feelings of anxiety and inadequacy, as well as a sense of being “child-like,” were especially pronounced in older individuals who lived in areas of social deprivation.

Our results also suggested that participants did not believe that digital health technologies could offer them anything of value, which is supported by existing literature indicating that people’s perception of the benefit they will gain from digital health technology determines their uptake [31]. These attitudinal factors can be related to the UTAUT concepts of “performance expectancy” and “effort expectancy,” [18], as well as the Digital Health Equity Framework, which defines beliefs about the potential help of digital health care as a key digital determinant of health [8]. Older participants living in areas of higher social deprivation often rejected the potential benefits of a more digitized health service on the basis that digital health care is impersonal and lacks a “human-touch.”

### Limitations

One limitation of our study was we only interviewed participants who were able to speak and understand English, which inevitably excluded some individuals from ethnic minority backgrounds. This may explain why we recruited mostly people from White British backgrounds. Belonging to an ethnic minority group, along with older age and social deprivation, increases the risk of experiencing kidney health inequalities [32]. Ethnic minorities are also disproportionately affected by digital exclusion [33] and are less likely to take part in health research for a variety of reasons [34]. This means that we missed the opportunity to capture the perspectives of a group of participants who may be the most excluded from engaging with digital health technologies, and to use these perspectives for informing future equitable digital health implementation strategies.

Secondly, we used the IMD to determine people’s socioeconomic status. However, because the IMD is based on people’s postcode, it only provides a proxy. Individual-level characteristics (such as income and educational attainment) might have been better indicators of social deprivation, given that some literature suggested that area-level deprivation indices are inconsistent indicators of individual-level social risks [35]. This means that some participants’ IMD scores may not have accurately reflected their actual level of social deprivation.

### Implications

Our research revealed that older people who are living in socially deprived areas might not have developed digital skills in their previous occupations, resulting in diminished digital literacy at retirement age. Therefore, government agencies and health care providers should provide mentoring opportunities to help improve overall digital literacy in this population, as well as support the involvement of caregivers. Existing research suggests that one-on-one support, from health care professionals, caregivers, or family members, is crucial to engaging older people in digital health technologies [31,36].

Providing caregivers with their own patient portal user account would enable them to act as a delegate and help vulnerable populations engage with these portals, especially those on a lower income [21]. Older people, particularly those living in areas of social deprivation, are often socially isolated [37]. Therefore, in addition to using family and friendship connections, efforts should be made to connect participants with appropriately outsourced resources and training to boost their engagement by, for example, providing one-on-one mentorship from a trained external supporter [28,38].

This study suggested that negative perceptions of age-related social norms about technology use were a barrier to engagement with digital health technologies. Government agencies that are responsible for digital health services need to implement strategies to help older people adjust to negative self-perceptions about their ability to learn new digital skills. Peer-to-peer support could be a key strategy to combat this low confidence and feeling “too old” to learn new digital skills. Such support includes programs to support people who are “newly converted” to digital health technology and want to share their skills with their peers [39], or peer support networks to build the skills of disabled older people living in a deprived area [40].

Older people living in socially deprived areas may lack intrinsic motivation to engage with digital health technologies due to a perception that these cannot offer any benefits that are relevant to them. This means researchers and technology developers should try to better understand how digital health technologies could add value for this group by, for example embedding participatory co-design into the development process [11,41], or implementing an inclusive design approach that centers on the experiences of disadvantaged and disinterested groups [27]. The Good Things Foundation in the United Kingdom produced evidence-based guidance for co-designing digital skills interventions for older people, which suggested that “digital champions” from the community, with appropriate support from local councils and charities, could help engage vulnerable populations [40]. In addition to inclusive design approaches, efforts should be made to promote available existing evidence of the value of digital health technologies.

Last, it is important to note that individuals who simply cannot or do not wish to engage with digital technology of any kind should not be forgotten. The provision of support to use digital health technologies is not enough to overcome all social and structural barriers that drive digital health inequities [42]. The King’s Fund therefore advises that health care services should always offer pathways with different levels of digital engagement so that service users always have the option of choosing a “low-tech” or “no-tech” pathway that offers face-to-face care [43].

While this study has contributed to the sparse body of literature on the factors that impact engagement with digital health technologies for older people who are socially deprived, further research is needed on the topic. Future studies should investigate the impact of individual-level characteristics that are related to social deprivation, such as income, educational attainment, occupational history, and ethnicity on older people’s engagement with digital health technologies.

### Conclusion

This study found that the key factors that hampered engagement in digital health technology by older people and those living in socially deprived areas were lack of opportunities in the workplace to become digitally proficient; lack of appropriate support from family and friends; negative perceptions of age-related social norms about technology use; and reduced intrinsic motivation to engage with digital health technology because of a perceived lack of relevant benefits. Lack of exposure to digital technology in the workplace and inadequate support to use digital technologies particularly impacted participants who were both older and socially deprived. These participants also felt significant anxiety around using digital technology and a sense of distrust toward digital health care on the basis that it is cold and impersonal.

Future digital health implementation strategies should make efforts to improve engagement in digital health technologies by older people and those living in socially deprived areas. This can be achieved by providing mentoring opportunities to help improve digital literacy; combating low digital confidence through peer-to-peer support; developing digital health technologies through participatory methods to better address the needs of older people and those who are socially deprived; and redesigning services to offer different levels of digitization, including “no-tech” pathways. This will help to ensure that all people with CKD and other long-term conditions can benefit from digitally enhanced health services, regardless of their age and socioeconomic background.

## Supplementary material

10.2196/60483 Multimedia Appendix 1 Supplementary materials.

## Abbreviations

CKD: chronic kidney disease
COREQ: Consolidated Criteria for Reporting Qualitative Research
IMD: Index of Multiple Deprivation
UTAUT: Unified Theory of Acceptance and Use of Technology
WHO: World Health Organization
