# Supplementary material for: Engagement With Digital Health Technologies Among Older People Living in Socially Deprived Areas: Qualitative Study of Influencing Factors
Source: JMIR Form Res. 2024 Dec 26;8:e60483. doi: 10.2196/60483 (PMC11694154; doi:10.2196/60483)
Supplement: Multimedia Appendix 1 [file formative-v8-e60483-s001.docx]

## **Supplementary table 1**

| **Participating hospital** | **Accredited Beds (N) [28]** |
| --- | --- |
| University Hospital Leuven | 1908 |
| University Hospital Gent | 1049 |
| General Hospital Groeninge | 1044 |
| OLV Hospital Aalst | 842 |
| General Hospital – AZ Sint-Lucas Gent | 779 |
| Antwerp Universtiy Hospital | 593 |
| General Hospital Maria Middelares | 542 |

## **Supplementary table 2**

| Hospital 1 | | | | | |
| --- | --- | --- | --- | --- | --- |
|  | # visits | Weight | | Height | |
|  |  | Completeness | Consistency | Completeness | Consistency |
| Surgical | 22634 | 96,71% | 99,77% | 33,92% | 99,64% |
| Paediatrics | 6109 | 98,77% | 99,70% | 95,58% | 99,69% |
| Geriatrics | 6615 | 98,72% | 99,07% | 59,47% | 99,62% |
| Hospital 2 | | | | | |
|  | # visits | Weight | | Height | |
|  |  | Completeness | Consistency | Completeness | Consistency |
| Surgical | 21495 | 97,94% | 99,75% | 41,86% | 99,84% |
| Paediatrics | 4970 | 99,09% | 99,55% | 97,26% | 99,96% |
| Geriatrics | 6662 | 98,57% | 98,90% | 41,07% | 99,89% |
| Hospital 3 | | | | | |
|  | # visits | Weight | | Height | |
|  |  | Completeness | Consistency | Completeness | Consistency |
| Surgical | 25880 | 46,43% | 98,98% | 39,52% | 98,91% |
| Paediatrics | 7797 | 52,24% | 97,99% | 31,54% | 96,83% |
| Geriatrics | 2531 | 88,42% | 98,57% | 25,56% | 99,69% |
| Hospital 4 | | | | | |
|  | # visits | Weight | | Height | |
|  |  | Completeness | Consistency | Completeness | Consistency |
| Surgical | 24784 | 91,46% | 99,55% | 90,82% | 99,68% |
| Paediatrics | 3482 | 89,43% | 99,78% | 12,84% | 99,11% |
| Geriatrics | 4475 | 77,97% | 98,71% | 77,07% | 99,33% |
| Hospital 5 | | | | | |
|  | # visits | Weight | | Height | |
|  |  | Completeness | Consistency | Completeness | Consistency |
| Surgical | 31961 | 92,59% | 99,51% | 44,67% | 99,36% |
| Paediatrics | 8356 | 94,89% | 98,39% | 77,24% | 99,66% |
| Geriatrics | 3485 | 91,79% | 98,44% | 62,35% | 99,59% |
| Hospital 6 | | | | | |
|  | # visits | Weight | | Height | |
|  |  | Completeness | Consistency | Completeness | Consistency |
| Surgical | 12905 | 93,79% | 99,51% | 96,99% | 99,57% |
| Paediatrics | 4090 | 97,53% | 99,60% | 11,29% | 99,57% |
| Geriatrics | 4391 | 96,74% | 97,65% | 87% | 99,53% |
| Hospital 7 | | | | | |
|  | # visits | Weight | | Height | |
|  |  | Completeness | Consistency | Completeness | Consistency |
| Surgical | 10179 | 94,46% | 99,07% | 94,07% | 99,36% |
| Paediatrics | 3611 | 97,66% | 96,97% | 94,99% | 99,47% |
| Geriatrics | 556 | 96,88% | 98,55% | 95% | 97,53% |

## **Supplementary table 3**

| **Hospital** | **Quote** |
| --- | --- |
| General Hospital Groeninge | “These results demonstrate the critical urgency to draw attention to the importance of data quality within the hospitals. A thorough data quality analysis should be part of each real-world-data project conducted with or within the hospital.” |
| OLV Hospital Aalst | “Data quality starts with gaining support from healthcare professionals to enter the right data in the right place. “ |
| Antwerp University Hospital | “A standard uniform data sharing agreement (DSA) is needed. This would ensure that data exchange for similar projects would be less complicated. Regarding the data quality results, it seems useful to perform a second use case taking into account parameters other than just weight and height and using additional dimensions for the analysis. |
